# Hydrogen Sulfide in Physiology and Diseases of the Digestive Tract

**DOI:** 10.3390/microorganisms3040866

**Published:** 2015-11-12

**Authors:** Sudha B. Singh, Henry C. Lin

**Affiliations:** 1 Section of Gastroenterology, Medicine Service, New Mexico VA Health Care System, Albuquerque, NM 87108, USA; E-Mail: sbsingh14@salud.unm.edu; 2 Division of Gastroenterology and Hepatology, Department of Medicine, the University of New Mexico, Albuquerque, NM 87131, USA

**Keywords:** Hydrogen sulfide, gastrointestinal tract, inflammation, Ischemia/reperfusion injury, motility, sulfate reducing bacteria, *Desulfovibrio*

## Abstract

Hydrogen sulfide (H_2_S) is a Janus-faced molecule. On one hand, several toxic functions have been attributed to H_2_S and exposure to high levels of this gas is extremely hazardous to health. On the other hand, H_2_S delivery based clinical therapies are being developed to combat inflammation, visceral pain, oxidative stress related tissue injury, thrombosis and cancer. Since its discovery, H_2_S has been found to have pleiotropic effects on physiology and health. H_2_S is a gasotransmitter that exerts its effect on different systems, such as gastrointestinal, neuronal, cardiovascular, respiratory, renal, and hepatic systems. In the gastrointestinal tract, in addition to H_2_S production by mammalian cystathionine-β-synthase (CBS), cystathionine-γ-lyase (CSE), H_2_S is also generated by the metabolic activity of resident gut microbes, mainly by colonic Sulfate-Reducing Bacteria (SRB) via a dissimilatory sulfate reduction (DSR) pathway. In the gut, H_2_S regulates functions such as inflammation, ischemia/ reperfusion injury and motility. H_2_S derived from gut microbes has been found to be associated with gastrointestinal disorders such as ulcerative colitis, Crohn’s disease and irritable bowel syndrome. This underscores the importance of gut microbes and their production of H_2_S on host physiology and pathophysiology.

## 1. Introduction

H_2_S is a notoriously toxic gas. Exposure to >700 ppm can cause sudden death. Occupational Safety and Health Administration (OSHA) exposure limit is 20 parts per million (ppm). Accidents and deaths following exposure to high concentrations of H_2_S have been documented [1,2,3,4]. Numerous reports have identified respiratory, cardiovascular, metabolic, neurological outcomes as a result of exposure to high levels of H_2_S. The most common sources of H_2_S include crude petroleum, hot springs, putrefying water and sewage plants. This gas is also produced by both vertebrates and invertebrates, in addition to prokaryotes and plants. Despite its lethal effects, H_2_S is a vital molecule for living organisms including humans. Abundant experimental evidences exist that indicate a role of H_2_S in physiology and pathophysiology. Many excellent reviews have discussed the biological roles of H_2_S with several focusing on its role in the gastrointestinal tract [5,6,7,8,9]. H_2_S is considered the third gasotransmitter, in addition to nitric oxide (NO) and carbon monoxide (CO), and is involved in inflammation, gut motility, oxidative stress, ulcer healing, vascular tone, neuromodulation, cryoprotection, memory formation, hormone secretion, apoptosis and many other vital biologic functions. Some of these processes are also targets for CO and NO although the mechanism of actions of these other gaseous signaling molecules may be different [10,11].

Based on its role in a wide range of organ systems, novel therapies are being attempted that aim at either suppressing H_2_S production or enhancing its availability [12]. H_2_S is found in many tissues, including the gastrointestinal (GI) tract, which is a major site of H_2_S production. H_2_S in the GI tract is produced not only by the enzymes CBS [13] and CSE [14] of the host but also by sulfate-reducing bacteria (SRB) that are resident microbes using the fermentation by-product hydrogen as the substrate. Normally, the production of this toxic gas is primarily compartmentalized to the colon since the majority of the gut microbial community is compartmentalized to the distal end of the gastrointestinal tract [15]. Recent progress in the studies of physiological function of H_2_S in the gut, with a special emphasis on bacteria-derived H_2_S is discussed in this review.

## 2. Sources of H_2_S

### 2.1. Mammalian H_2_S

**Figure 1:**
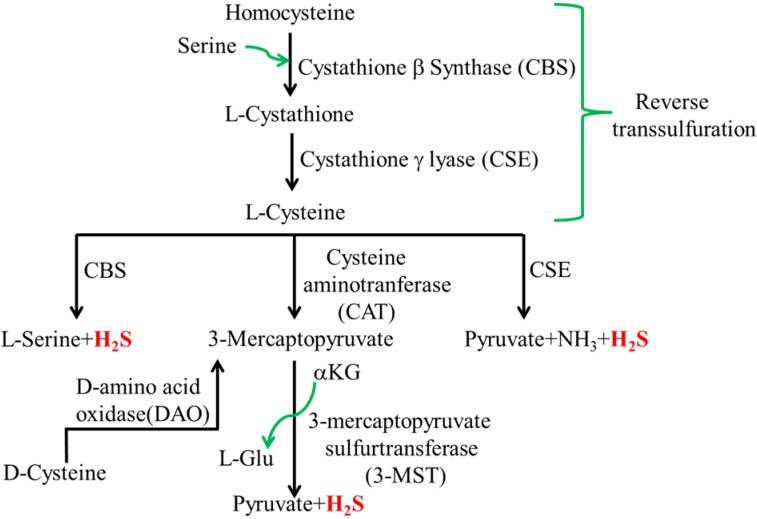
Biosynthesis of H_2_S by mammalian cells.

Endogenous H_2_S is found in various tissues such as central nervous system, liver, kidney, cardiovascular system, lungs and gastrointestinal tract. H_2_S is generated enzymatically through the reverse transsulfuration pathway by the activity of cystathionine-β-synthase (CBS), cystathionine-γ-lyase (CSE), and 3-mercaptopyruvate sulfurtransferase (3MST) [16,17,18] (Figure 1). Non-enzymatic pathways of H_2_S generation exist and reduce elemental sulfur to H_2_S [19]. However, these pathways only account for a small quantity of H_2_S produced in the mammalian tissues. CBS and CSE produce H_2_S using l-cysteine and homocysteine as substrates. 3-MST does so via the intermediate synthesis of 3-mercaptopyruvate, which is produced by cysteine aminotranferase (CAT) using cysteine as a substrate.

A fourth pathway also exist that utilizes d-Cysteine as a substrate and involves enzymes d-amino oxidase and 3-mercaptopyruvate sulfurtransferase specifically in the cerebellum and kidney [20]. CBS and CSE are found in the cytosol while 3-MST is found in mitochondria as well as cytosol [21]. CBS, CSE and CAT use pyridoxal 5′-phosphate (PLP) as a cofactor, whereas 3-MST uses zinc as a cofactor. CBS and CSE are differentially expressed in various organs, such as the liver, kidney, brain, intestine, heart, lung [22,23,24], and in various cell types, such as interstitial cells of Cajal (ICC), enteric neurons [25] and smooth muscle cells [26]. By and large, CBS is predominant in the nervous system and CSE in the cardiovascular system. MST has been detected in hippocampal pyramidal neurons [16] cardiomyocytes, glial cells, kidney cells, liver cells and vascular smooth muscle cells [21,27].

### 2.2. Intestinal H_2_S Production by Resident Microbes

H_2_S in the intestine is also produced by some members of the resident gut bacterial community. Analysis of fecal H_2_S revealed that 50% of fecal H_2_S is derived from bacteria as evident by the dependence of only 50% H_2_S synthesis on pyridoxal 5′-phosphate(P-5-P*)* and that in germ free mice, fecal H_2_S synthesis is only 50% of that in observed in colonized mice [28]*.*

Microbial communities are an integral part of human gastrointestinal tract and carry out vital functions such as digestion, production of vitamins and other nutrients, resistance to colonization by invading pathogens and mucosal immunity [29,30]. Bacterial fermentation of complex carbohydrates in the colon releases large amounts of hydrogen, which is consumed by hydrogenotrophs that include methanogens, acetogens or sulfate-reducing bacteria, which produce methane, acetate, or H_2_S, respectively. Methanogens and SRB compete for H_2_ and, thus, usually one of these hydrogenotrophs predominates in the large intestine. When methanogenesis is the dominant pathway for handling fermentation derived H_2,_ methane appears in the exhaled breath (methane excretor).

**Figure 2:**
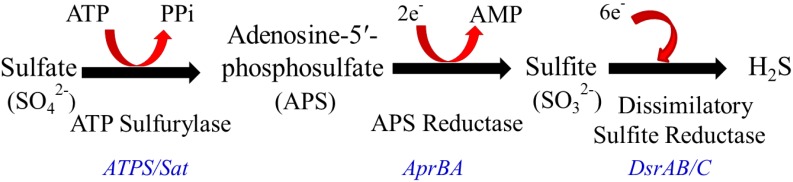
Dissimilatory sulfate reduction by SRB. Sulfate is activated to adenosine-5-phosphosulfate (APS) by ATP sulfurylase (ATPS), also known as sulfate adenylyltransferase (Sat). The second enzyme, APS reductase (Apr), converts APS to sulfite, which is reduced to sulfide by dissimilatory sulfite reductase (Dsr).

Among the most prominent producers of H_2_S are sulfate-reducing bacteria (SRB) [31,32]. On the basis of the prevalence of methane excretors, sulfate reduction is the dominant hydrogenotrophic pathway in ~60% of humans. Enumeration of SRB by culture dependent methods revealed a range of 10^3^–10^11^ bacteria per gm of human feces [33]. SRB belong to the class δ-Proteobacteria and produce H_2_S using the enzyme complex dissimilatory sulfite reductases (DSR) (Figure 2). The most predominant genus in this category is *Desulfovibrio*, which utilize lactate and hydrogen as substrate. Other genera being *Desulfobacter*, *Desulfomonas*, *Desulfobulbus*, and *Desulfotomaculum*. The SRB use sulfate as a terminal electron acceptor for respiration, with the concomitant production of H_2_S [15]. Other bacterial species belonging to different genera, such as *Streptococcus*, *Fusobacterium*, *Salmonella*, *Enterobacter*, and *Helicobacter* also produce H_2_S from l-cysteine, by the activity of cysteine desulfhydrase [34,35,36,37,38]. An excellent review by Carbonero, *et al.* summarizes microbial pathways of sulfur metabolism in the colon [39].

Colonic bacteria are responsible for generating vast quantities of H_2_S. Up to 1000 ppm of H_2_S has been detected in the rat cecum [40]. Analysis of flatus composition of human subjects revealed H_2_S concentration in the range of 0.2 to 30 ppm [41]. A high level of H_2_S induces DNA damage, inhibits cytochrome c oxidase, and inhibits butyrate oxidation. Weisiger *et al.* reported comparable thiol S-methyltransferase activity in colonic mucosa and liver [42] where methylation of H_2_S was the suggested mechanism for detoxification of H_2_S. The rate of methylation of H_2_S was found to be much lower (10^−13^ mol/min per milligram) and could not account for the rapid detoxification of H_2_S. Moreover, metabolism of H_2_S was found to be unresponsive to *S*-adenosyl-l-homocysteine, an inhibitor of S-methyltransferases [43]. Later, it was found that oxidation of H_2_S to thiosulfate was an efficient mechanism by which colonic mucosa dispose off this toxic gas [44]. It was observed that H_2_S was metabolized to thiosulfate by cecal and right colonic mucosa at a rate that was about twenty times greater than that of the gastric or small bowel mucosa and eight times greater than that of the liver [41]. H_2_S is also excreted out as flatus or in feces.

## 3. Impact of SRB, Bacterial Derived H_2_S and Lipopolysaccharide (LPS) on Host

### 3.1. SRB and Inflammatory Diseases

Gut microbes control many aspects of human physiology and are key players in health and disease. An imbalance in the number or composition of gut microbes (dysbiosis), which can be triggered by disruptors such as antibiotics, stress, Western diet and infection, has been linked to a plethora of diseases such as obesity [45], Inflammatory bowel disease (IBD) [46], Irritable bowel syndrome (IBS) [47,48], cardiovascular diseases [49], type 2 diabetes [50] and cancer [51]. Dysbiosis tips the delicate microbial balance and often accompanies intestinal inflammation. This altered environment may promote opportunistic growth of Gram-negative members of families *Enterobacteriaceae* and *Desulfovibrionaceae.* Moreover bacteria, such as SRB, are resistant to broad spectrum antibiotics [52] as bacterial H_2_S is a defense mechanism against antimicrobials [53]. This characteristic of SRB may favor a bloom of these bacteria in the setting of repeated antibiotic use.

A high number of SRB have been found in patients with ulcerative colitis (UC) [54]. SRB was also observed in higher number in feces of IBD Crohn’s Disease (CD and UC) patients [55]. In addition, fecal H_2_S levels were reported to be greater in UC compared to controls [56,57]. Interestingly, consumption of meat and high sulfur or sulfate containing diet that could promote the production of H_2_S was associated with an increased likelihood of relapse for UC patients further suggesting a role of these microbes in the pathogenesis of UC [58]. 5-aminosalicylic acid (5-ASA), an anti-inflammatory medication commonly prescribed for UC, also inhibits SRB growth and production of H_2_S suggesting an additional explanation for its efficacy [59]. H_2_S inhibits oxidation of *n*-butyrate in colonic epithelial cells, thus compromising barrier function by inducing an energy deficit [60]. This may explain the increase in intestinal permeability in UC [61]. These studies also suggested that SRB growth may be favored under inflammatory conditions. However, other reports show that fecal H_2_S [62] and SRB [57] were not elevated in UC. Moreover, blocking bacterial H_2_S by bismuth did not show an improvement in the mouse model of dextran sulfate induced colitis [63]. However, this model of chemical colitis only partially reproduce the phenotype seen in ulcerative colitis in humans.

**Figure 3:**
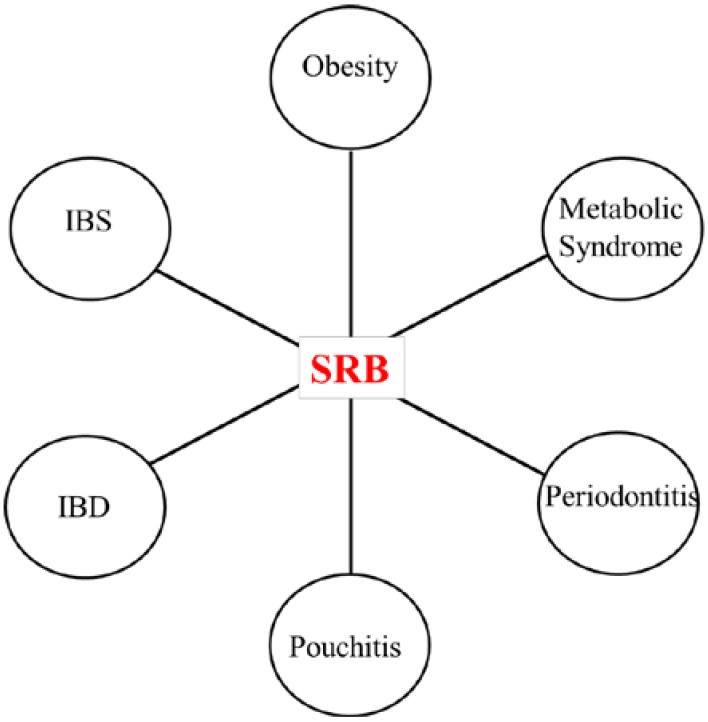
Association of SRB with human diseases. The evidence linking SRB with diseases is inconclusive.

SRB were also found to be associated with other inflammatory conditions such periodontitis [64] and increased levels of H_2_S have also been detected in periodontal pockets [65]. SRB and H_2_S were also found to be much higher in Pouchitis [66]. SRB were found in greater number in constipation-predominant IBS patients compared to controls [67]. *Desulfovibrionaceae* were also found to be higher in number in animal models of metabolic syndrome [68]. Figure 3 summarizes the association of SRB with diseases. In addition, several case studies have reported *D. desulfuricans* bacteremia under different clinical conditions [69,70,71]. Some studies have highlighted the effects of probiotic and prebiotic treatments on SRB population in the gut. In one report, 4 weeks of oral consumption of probiotic *Lactobacillus plantarum* P-8 *Lp*-8 treatment caused a significant reduction of *Desulfovibrio* genus in human volunteers [72]. In another study, mice fed with the prebiotic glycomacropeptide (GMP) showed a dramatic reduction in fecal load of *Desulfovibrio* and also reduced levels of proinflammatory cytokines IFN-γ, TNF-α, and IL-1β [73].

In another diet-based analysis, when obese subjects were given diets to balance their gut microbes, weight loss was accompanied by a decrease in H_2_S-producing families *Desulfovibrionaceae* and *Enterobacteriaceae (Escherichia*, *Shigella*, *Klebsiella*, and *Citrobacter*), along with a decrease in proinflammatory Tumor necrosis factor (TNF-α) and Interleukin (IL-6) cytokines [74]. There are conflicting reports including another study showing that *Desulfovibrio* levels were much lower in obese/overweight children compared to controls [75]. Thus, the role of SRB and SRB derived H_2_S in the pathogenesis of a disease condition, such as obesity, remain unclear. Even as mounting evidence suggests that SRB is associated with inflammatory conditions, it is not clear whether this is an epiphenomenon or SRB are the etiological agents of the disease.

In addition to SRB, other H_2_S-producing gut bacteria, such a *Fusobacterium*, have also been found to be associated with IBD [76]. *Fusobacterium* isolated from IBD patients induced inflammatory responses in colonic cell lines [77]. Genomic analysis of the colorectal carcinomas revealed the expansion of *Fusobacterium* on the mucosal surface of these cancers [78]. Since H_2_S is a known genotoxic agent [79], it is plausible that increased H_2_S production as a result of expansion of *Fusobacterium* may be the link between *Fusobacterium* and colorectal tumors. Moreover, *E. coli*, another H_2_S-producing enteric bacteria, has also been found to be associated with CD and colorectal cancer [80]. The connection between H_2_S-producing gut bacteria and inflammation was further supported by the finding that *Bilophila wadsworthia*, a sulfite-reducing bacteria that generates H_2_S similar to *Desulfovibrio* was higher in concentration in mice fed with high fat diet and this was associated with proinflammatory responses in genetically susceptible mice [81].

H_2_S production by these gut microbes can also lead to secondary effects including promoting the growth of pathogens. H_2_S in the gut is generally detoxified to thiosulfate by the colonic mucosa. Under the conditions of inflammation, reactive oxygen species (ROS), generated by the activity of neutrophils, oxidize thiosulfate to tetrathionate. *Salmonella* is capable of readily utilizing tetrathionate as electron acceptor and this confers growth advantage for this enteric pathogen [82] in overcoming colonization resistance. Moreover, *Salmonella* also produces H_2_S from thiosulfate [83,84]. Thus, H_2_S production by these bacteria may allow positive feedback favoring growth of pathogens such as *Salmonella*.

### 3.2. Effects of SRB Activity on Host

What is the effect of SRB and other H_2_S-producing gut microbes on host physiology and pathophysiology? Are SRB beneficial or harmful? Can this information be utilized to develop novel therapies to tackle inflammatory diseases based on targeting SRB?

Studies on effect of LPS derived from SRB provide further clues about potential impact of these bacteria on the host. LPS from *D. desulfuricans* causes secretion of proinflammatory cytokine IL-6 and neutrophils, basophils, and T-cell attracting chemokine IL-8 by human gingival fibroblast (HGF-1) cell line [85] and in human umbilical vein endothelial (HUVEC) cells [86] and IL-6 by Tamm–Horsfall protein 1 (THP1) cells [87], whereas it downregulated IL-8 secretion by Colonic adenocarcinoma (Caco-2 cells) [88]. This inconsistency in these results could be due to the fact that Caco-2 cells are derived from colon, which is a natural habitat for SRB and thus may respond differently to these bacteria or its products. It would be valuable to study if *Desulfovibrio*-derived LPS triggers the production of mammalian or bacterial H_2_S. Interestingly, *E.coli* LPS can cause increase in plasma H_2_S levels in mice along with inflammation and increased expression of CSE [89].

However, the direct impact of SRB on host physiology remains unknown. One possible way to study the unilateral effects of SRB on the host is by introducing exogenous SRB into the host. One such study was conducted by Rey *et al.*, where gnotobiotic mice were colonized with gut bacterial species with or without *Desulfovibrio piger* and these mice were fed different diets [90]. High levels of cecal H_2_S were observed in mice colonized with *D. piger* compared to mice without *D. piger.* In addition, there was a decrease in the host consumption of amino acids in mice inoculated with *D. piger*. Mice colonized by *D. piger* had lower mRNA levels of several Ig subclasses such as IGHM and IGHA1 and claudin-4 (a tight junction protein) but high levels of matrix metalloproteinase-7; however, there was no significant effect on the gut barrier integrity. Another study highlighted the role of SRB in the biotransformation of environmental toxicants such as bacteria-driven thiolation of arsenic that enters the gut through food contaminated with this carcinogen. This activity correlates with the production of H_2_S by SRB [91].

Oral administration of bacteria into the host is a standard protocol to study the host microbe relationship and has been utilized to understand the effect of probiotics [92,93] and pathogens [94,95] in animal models. Thus, oral administration of SRB in rodents may be used as an important tool to understand the impact of SRB and its production of H_2_S on physiological functions of the host.

## 4. Effects of Host-Derived H_2_S on the Gastrointestinal System

Table 1 summarizes some of the commonly studied functions of H_2_S in gut physiology and the diseases it is associated with. Most of the physiologic studies on H_2_S have been carried out by either manipulating the endogenous mammalian source of H_2_S such as CBS and CSE by gain or loss of function or by addition of exogenous donors of H_2_S, such as sodium hydrosulfide (NaHS), and slow releasing donors, such as GYY4137. Some of these functions are discussed below.

**Table 1:** H_2_S in gut physiology and diseases.

Physiology	Disease
Protection against I/R Injury	Inflammatory Bowel Disease
Inflammation (Pro-/Anti-inflammatory)	Colitis
Motility (Excitation/Inhibitory)	Pouchitis
Nociception (Pro-/Anti-nociceptive)	Periodontitis
Colonic Secretion	Halitosis
Ulcer Healing	Sepsis
Antioxidant	Obesity
Apoptosis	

### 4.1. H_2_S in Ischemia/Reperfusion Injury

Ischemia followed by reperfusion (I/R) is a commonly occurring pathological condition that can lead to tissue and organ damage. Several clinical events are almost universally associated with some degree of I/R injury including bypass surgery, stroke, organ transplantation and myocardial infarction. Several studies have provided evidence for a protective role of H_2_S in I/R injury. In the heart, both *in vivo* and *in vitro* studies using endogenous and exogenous sources of H_2_S have highlighted the protective role of H_2_S on myocardial I/R. [96,97,98] that appears to involve autophagy [99,100]. Similarly, studies have shown that H_2_S protects against hepatic [101,102,103] renal [104,105,106] and brain I/R injury [107,108].

In the intestine, I/R injury can result in multiorgan failure and systemic inflammation and is associated with significant mortality and morbidity [109]. A handful of studies have confirmed the role of H_2_S in I/R injury. Liu *et al.* demonstrated that an H_2_S donor, NaHS protected rats from severe intestinal I/R injury by increasing the activity of antioxidant enzymes superoxide dismutase and peroxidase [110,111,112,113]. In another study, Pan *et al.* showed that NaHS protected rats from intestinal I/R injury in response to cardiac arrest followed by cardiopulmonary resuscitation (CPR). Protective effects of NaHS may be mediated through reduction of reactive oxygen species (ROS), inhibition of inflammation, apoptosis and HIF-1α activation. [114]. Furthermore, H_2_S protected enterocytes from hypoxia as evidenced by reduced apoptotic index [115]. H_2_S protected against injury 1 h and 2 h after ischemia and preserved the crypt villi height. H_2_S donor added 24 h prior to I/R reduced post ischemic intestinal mitochondria dysfunction and this was mediated by calcium-activated big potassium (BK_Ca_) channels [116]. In addition, preconditioning of the small bowel with the exogenous H_2_S donor NaHS inhibited leukocyte rolling (LR) and adhesion (LA) induced by I/R through a p38 MAPK-dependent mechanism [117]. Thus, protective effects of H_2_S on I/R injury of different organs are mediated largely by its antioxidant, anti-inflammatory and anti-apoptotic properties. A detailed review of role of H_2_S in I/R injury is discussed in an excellent review by Wu *et al.* [118].

### 4.2. H_2_S in Intestinal Inflammation

Reports of the role of H_2_S in inflammation are, at times, diametrical. H_2_S can have a pro-inflammatory or anti-inflammatory effects depending on what model system is used, the source of H_2_S (exogenous *versus* endogenous), the bioavailability of H_2_S (slow *versus* fast release H_2_S donors) and the route of administration. The effects of these factors are exemplified by reports in experimental models of sepsis induced by cecal ligation and puncture (CLP). Administration of NaHS (~5.6 mg/kg) subcutaneously improved survival in the experimental C57BL6/J mouse model of sepsis before and after the onset of CLP, decreased TNF-α and IL-10 cytokine production, decreased caspase-3 activation, increased bacterial clearance and decreased expression of CHOP [119]. In contrast, in a study by Zhang *et al.* in Swiss mice, CLP-induced systemic inflammation was associated with increased plasma levels of H_2_S and higher levels of CSE gene expression and treatment with NaHS (10mg/kg i.p.) further exacerbated the CLP-induced inflammation while administration of DL-propargylglycine (PAG; 50 mg/kg i.p.) an inhibitor of CSE, reversed these effects [120]. A follow-up study by Zhang *et al.* demonstrated that the pro-inflammatory effect of H_2_S donor NaHS (10mg/kg i.p.) on CLP associated sepsis depended on nuclear factor kappa B (NF-kB) activation [121]. Treatment with PAG (50 mg/kg i.p.) decreased IL-1β, IL-6, and TNF-α. When a higher dose of NaHS was used intraperitoneally rather than subcutaneously, a clear pro-inflammatory effect of H_2_S (NaHS; 14 μmol/kg i.p.) could be shown in a LPS-induced model of endotoxic shock in male Swiss mice where PAG (50 mg/kg i.p.) reduced this effect [89]. Thus, it appears that dosage and route of administration of NaHS may dictate whether the effects of NaHS will be pro- (high dose, i.p.) or anti-inflammatory (low dose, subcutaneous). The proinflammatory effect mediated by H_2_S may be dependent on the extracellular signal-related kinases (ERK) pathway [122]. Once again, in this study, NaHS was administered by i.p. ERK dependent pro-inflammatory effects of H_2_S were also observed in human monocytic cell line U937 [123].

Another factor that may influence the beneficial *versus* deleterious role of H_2_S in inflammation is the choice of H_2_S donor, which may determine bioavailability of H_2_S. While many reports tested the effect of the fast releasing H_2_S donor NaHS, some studies have tested the effect of H_2_S using a slow releasing donor GYY4137 (morpholin-4-ium-4-methoxyphenyl (morpholino) phosphinodithioate), which has been shown to exert an anti-inflammatory action in endotoxic shock model in rats [124]. GYY4137 (50 mg/kg, i.p.) administered to rats following LPS treatment decreased the rise in plasma proinflammatory cytokines (TNF-α, IL-1β, IL-6) and lung myeloperoxidase activity, increased plasma concentration of the anti-inflammatory cytokine IL-10, and decreased tissue damage [124]. Anti-inflammatory effects of GYY4137 are more consistently reported across different experiments [125,126,127]. GYY4137 treatment may be physiologically more relevant than using NaHS as this chemical may mimic slow and sustained release of endogenous H_2_S by cellular enzymes. In comparing the effects of fast releasing NaHS *versus* slow releasing GYY4137 on LPS treated cultured macrophages, it was found that while GYY4137 clearly and dose-dependently reduced LPS evoked expression of proinflammatory cytokines, the results with NaHS were biphasic [128]. At low concentrations, NaHS did not exhibit any effect on proinflammatory cytokines but at higher concentration, was pro-inflammatory by causing enhancement of LPS-induced expression of IL-1β and TNF-α. In addition, treatment of LPS-exposed RAW 264.7 cells with GYY4137 caused a concentration-dependent inhibition of the activation of NF-kB, whereas NaHS inhibited NF-kB at high concentrations while promoting NF-kB activation at lower concentrations. This study gives additional insight into the discrepancies observed among different H_2_S donors.

Several other pathways have been reported to explain the anti-inflammatory effects of H_2_S. H_2_S showed a protective effect against IFN-γ and TNF-α induced injury of epithelial barrier function in Caco2 cells by blocking NF-kB p65 activation [129]. H_2_S also prevented NF-kB activation, apoptosis and inflammation upon ischemia/reperfusion I/R injury of the gastric epithelial cells [130]. Another study reported that IL-10 may promote H_2_S synthesis in the colon which, in turn, exerts a protective effect. Specifically, IL-10 deficient mice fed a vitamin B-deficient diet had low levels of colonic H_2_S and an increased level of homocysteine in the serum and exhibited severe colonic inflammation [131]. Administration of IL-10 to the IL-10-deficient mice restored colonic H_2_S synthesis and significantly decreased serum homocysteine levels. H_2_S donors reduced colitis and H_2_S inhibitors exacerbated colitis [132,133]. 5-amino-2-hydroxy-benzoic acid 4-(5-thioxo-5H-[1,2]dithiol-3-yl)-phenyl ester hydrochloride ATB-429, a H_2_S releasing drug has been shown to reduce the severity of colitis [134]. Flannigan *et al.* reported that protective effects of H_2_S in experimental colitis are mediated via stabilization of hypoxia-inducible factor (HIF)-1α and that the loss of CSE-derived H_2_S production led to decreased HIF-1α stabilization and exacerbation of colitis [135].

In a recent study by Motta *et al.*, H_2_S promoted resolution of colonic inflammation by facilitating microbial biofilm and mucus production. Therapeutic delivery of H_2_S into a hapten-induced colitis mouse model reduced inflammation, restored the microbial biofilm, and increased the production of mucus granules [136]. One mechanism by which H_2_S resolves inflammation is by reducing leukocyte migration to the site of injury [137] and by enhancing neutrophil apoptosis [138]. In addition, application of H_2_S to human or murine macrophages increased chemotaxsis of macrophages and the rate of phagocytosis of the bacterium *Escherichia coli* which resulted in a reduction of the accumulation of inflammatory cells [139]. In addition, H_2_S also promotes the activity of cyclooxygenase (COX)-2 [140], an enzyme that plays crucial role in ulcer healing and resolution of inflammation [141]. Another mechanism by which H_2_S exerts its anti-inflammatory effect is by involving annexin-A1 pathway [142]. Following the onset of inflammation the l-cysteine/H_2_S pathway contributes to trigger AnxA1 mobilization, which in turn controls leukocyte trafficking. As discussed in an earlier section, H_2_S protects against I/R injury and ulcer healing via its anti-inflammatory properties. Thus, based on the existing evidence, it is tempting to assume that H_2_S is generally anti-inflammatory. However, more physiologically relevant studies are needed.

### 4.3. H_2_S in Ulcer Healing

Wallace and colleagues showed that gastric H_2_S synthesis was increased following induction of ulcer in rat stomach [143]. Administration of l-cysteine, a precursor for H_2_S synthesis, as well as exogenous H_2_S donors significantly enhanced gastric ulcer healing, whereas the administration of PAG, an inhibitor of endogenous H_2_S synthesis, impaired ulcer healing [143]. A recent study indicated that pretreatment with l-cysteine, vitamin B_6_ (a cofactor for the enzyme CBS) and NaHS protected the gastric mucosa against ethanol-induced gastric lesions [144]. This study corroborated previous findings by Medeiros *et al.* who demonstrated that administration of PAG reversed gastric protection induced by l-cysteine in an ethanol-induced gastric damage model [145]. However, Chávez *et al.*, presented contradictory findings where PAG treatment conferred protection to the gastric mucosa in ethanol-induced gastric injury [146]. It is possible that the discrepancy in their results is due to the concentration (absolute *versus* 50% ethanol in the other two studies) and timing of ethanol introduction following PAG (3 h in Chávez *et al. versus* 30 min in Medeiros *et al.*) and NaHS administration (2 h *versus* 30 min). It is also possible that under these different settings, other H_2_S independent protective pathways may come into play, such as glutathione which is also modulated by l-cysteine and NaHS [145,147]. Nevertheless, Chávez *et al.* did show the protective effects of l-cysteine on gastric mucosa in their model of ethanol-induced gastric injury. The protective role of H_2_S on gastric ulcer may be facilitated by its anti-inflammatory effects since NaHS and l-cysteine treatment decreased gastric lesions along with a decrease in mRNA and plasma levels of IL-1β and TNF-α [148]. In another study, PAG increased gastric mucosal ulceration and the number of hemorrhage sites following gastric mucosal damage induced by I/R injury and this effect was attenuated by administration of l-cysteine via inhibition of free radical generation [149]. It was shown that H_2_S degradation was decreased at the site of ulceration and that local high concentration of H_2_S may be involved in the repair [150]. In a rat model of water immersion and restraint stress induced gastric ulcers, exposure to H_2_S reduced gastric ulcer index and also downregulated the expression of GRP78 and caspase 12, markers of endoplasmic reticulum stress [151,152]. Administration of ACS14, a H_2_S-releasing derivative of aspirin, caused attenuation of gastric damage induced by aspirin. ACS14 also attenuated Aspirin-suppressed superoxide dismutase-1 (SOD-1) expression and GSH activity [153]. Nonsteroidal anti-inflammatory drugs (NSAID) and acetyl salicylic acid (ASA) decreased H_2_S formation, CSE expression and induced gastric injury, increase in myeloperoxidase (MPO) and TNF-α [154]. Treatment with NaHS significantly reduced these effects whereas treatment with PAG exacerbated gastric injury induced by NSAID. These effects of H_2_S against gastric injury were mediated through activation of K_ATP_ channels since glibenclamide, (K_ATP_ channel blocker) reversed effect of NaHS and pinacidil (a K_ATP_ opener) attenuated gastric mucosal injury. H_2_S regulates processes such as inflammation, apoptosis, nociception and I/R injury by activating K_ATP_ channels. While it is not clear how H_2_S activates K_ATP_ channels, it has been found that K_ATP_ channel is sulfhydrated by H_2_S [155] and that H_2_S activated cloned rvKir6.1/rvSUR_1_ channels by interacting with extracellular cysteine residues.

### 4.4. H_2_S in Intestinal Motility

Experimental evidence generally supports an inhibitory role of H_2_S on gastrointestinal motility [156,157,158,159,160,161]. The negative effect of H_2_S in some cases is mediated through K_ATP_ channels [159,162] but in others, blocking of K_ATP_ channels did not affect H_2_S-induced inhibition [158,163]. The relaxant effect of H_2_S on colonic motility has also been explained, in part, by its direct inhibition of l-type calcium channel. H_2_S inhibits both l-type calcium channels and BK_Ca_ channels in smooth muscle cells of rat colon [164]. In vascular smooth muscle cells, opening of K_ATP_ channels hyperpolarizes cell membrane and inactivates voltage-dependent l-type Ca^2+^ channels, leading to relaxation of smooth muscle cell and dilation of blood vessel. Recently, it was demonstrated that H_2_S exerted suppressive effects on colonic contractility by decreasing neurally mediated cholinergic and tachykinergic excitatory pathways [165]. It has been previously shown that H_2_S significantly reduced cholinergic mediated contractions [158,160]. In another study, NaHS inhibited the contractile activity of smooth muscle cells in rat stomach and jejunum [165]. l-cysteine, a precursor of H_2_S, inhibited the electrical stimulation (ES) induced contraction of mouse ileum. Contractions increased following the administration of aminooxyacetic acid, an inhibitor of CBS and CSE. The treatment with glibenclamide, a K_ATP_ channel blocker, reduced both the l-cysteine response and NaHS-induced inhibition of contractions [166]. The same group later identified that inhibition of electrical field stimulation-induced contractions in ileum by l-cysteine and d,l-homocysteine was decreased by the treatment with 2-aminobicyclo [2.2.1]heptane-2-carboxylate (BCH), an inhibitor of l type and B°^+^ transporter systems, thus highlighting the role of the amino acid transport system in l-cysteine regulation (via H_2_S) of motility [167].

A dual excitatory and inhibitory role of H_2_S was demonstrated in smooth muscle contractility, which was dependent on the dose of NaHS. Typically at lower concentrations, NaHS stimulated the contraction while at higher doses caused relaxation. NaHS also caused a biphasic effect, early transient excitation and late long-lasting inhibition on the motility of rat duodenum jejunum, ileum and colon [168]. The excitatory effects of NaHS were mediated by TRPV1 channels since capsazepine, a TRPV1 antagonist, inhibited this effect. TRPV1 channels are known to be activated by NaHS [169,170] Glibenclamide, on the other hand, did not affect NaHS induced excitatory effect but rather inhibited NaHS-induced long-lasting inhibition on the contraction of muscle strips, indicating the role of K_ATP_ channels.

## 5. Concluding Remarks

Over the last two decades or so, studies have uncovered many roles of H_2_S in physiology and disease. H_2_S has been found to have dichotomous effects (stimulatory and inhibitory) on several gastrointestinal processes such as inflammation, contractile responses, nociception, cancer and apoptosis. Thus the biological functions of H_2_S remain rather confusing and controversial. However, some effects of H_2_S appear to be unequivocal. H_2_S has been found to be protective in several animal models of I/R injury in the gastrointestinal tract, brain, lung, kidney and heart. Experimental evidences also point towards the overall protective effects of H_2_S in colitis via its anti-inflammatory properties. ATB-429, an H_2_S releasing derivative of mesalamine, seems promising in the treatment of colitis. H_2_S also protects against gastric injury induced by administration of NSAIDS. Similarly, several other H_2_S releasing compounds are effective in cancer [171] and arthritis models [172]. Thus, although excessive H_2_S may be a contributing factor in diseases, the overarching effect of H_2_S appears to be beneficial.

Owing to its role in several organs systems, tissues and cells, the biological significance of H_2_S has now been recognized and several H_2_S releasing compounds are being tested in clinical trials for the treatment of human disorders. However, our knowledge of this important gasotransmitter is far from complete. Interestingly, the multifaceted roles of H_2_S parallel those of other major gaseous signaling molecules NO and CO. All three regulatory gaseous molecules are synthesized from both the mammalian and microbial sources in the body. All three are hazardous at high concentrations but favorable at low concentrations. In fact, all three gases share many common chemical properties and exert similar biological effects albeit via different mechanisms. Moreover, H_2_S, CO and NO cross talk and stimulate or inhibit each other to regulate several physiological functions. Given several similarities between H_2_S, CO and NO in terms of their properties and physiological function, it may be helpful to exploit the lessons learnt from NO and CO to investigate the functions of H_2_S and to gain a better understanding of its role in health and disease and to develop H_2_S based therapies.

Lastly, it is important to emphasize the crucial role of gut microbes in host physiology. The impact of bacteria-derived H_2_S on host physiology and pathophysiology is largely unexplored but crucial area of research. Much work needs to be done to identify the role of bacteria derived H_2_S on host biological processes. Understanding how bacteria-derived H_2_S may impact host biology can help us gain a better understanding of underlying mechanism of diseases such as IBS, IBD, and obesity.
